# Strain-dependent interactions of *Streptococcus gallolyticus* subsp. *gallolyticus* with human blood cells

**DOI:** 10.1186/s12866-017-1116-1

**Published:** 2017-10-27

**Authors:** Imke Grimm, Melanie Weinstock, Ingvild Birschmann, Jens Dreier, Cornelius Knabbe, Tanja Vollmer

**Affiliations:** Institut für Laboratoriums- und Transfusionsmedizin, Herz- und Diabeteszentrum Nordrhein-Westfalen, Universitätsklinikum der Ruhr-Universität Bochum, Bad Oeynhausen, Germany

**Keywords:** *S. gallolyticus* subsp. *Gallolyticus*, Infective endocarditis, Platelet aggregation, Whole blood assay, THP-1 monocytes, Phagocytosis

## Abstract

**Background:**

*Streptococcus gallolyticus* subsp. *gallolyticus* (*S. gallolyticus)* is the causative pathogen in up to 20% of streptococcal-induced infective endocarditis (IE) cases. However, the underlying mechanisms of pathogenesis in *S. gallolyticus* have not yet been solved. Pathogens causing IE need to employ virulent strategies to initiate and establish infections, such as escape the bloodstream, invade the host-cell, and persist intracellularly. In this study, we examined the induction of inflammation by different *S. gallolyticus* strains in relation to their survival in whole blood and cell culture models as well as their ability to induce platelet aggregation. Phagocytosis of these bacteria by macrophages, followed by intracellular survival, was also quantified.

**Methods:**

In whole blood and THP-1 cell culture assays bacterial growth kinetics was determined by plating, followed by colony counting. Induction of interleukin (IL)-6 expression in whole blood of three healthy volunteers, caused by different strains, was quantified by ELISA. Gene expression of cytokines (*IL1B, IL6 and IL8*) was quantified by real-time PCR after stimulating THP-1 monocytes with bacteria. Induction of platelet aggregation was analyzed by light transmission aggregometry using the BORN method. A macrophage model was used to analyze phagocytosis of strains and their survival in macrophages within 48 h.

**Results:**

Strains promoted IL-6 secretion in a time-dependent fashion. For example, DSM16831 induced IL-6 secretion in whole blood earlier than other isolates, and was eliminated in the whole blood of one volunteer, whereas UCN34 could grow. Platelet aggregation depended on the different isolates used and on the individual platelet donor. Two strains (AC1181 and 010672/01) induced cytokine gene expression in THP-1 monocytes only marginally, compared to other strains. The phagocytosis rate of *S. gallolyticus* isolates differed significantly, and the isolates UCN34 and BAA-2069 could persist for a considerable time in the phagocytes.

**Conclusion:**

The strain-dependent differences of *S. gallolyticus* isolates, observed during interaction with human blood cells, support the hypotheses that divergences in individual virulence factors determine a distinct pathogenicity of the isolates. These data constitute an additional step towards the elucidation of mechanisms in the complex, multifactorial pathogenesis of this IE pathogen.

## Background

*Streptococcus gallolyticus* subsp. *gallolyticus* (formerly *Streptococcus bovis* biotype I) is a frequent pathogen in infective endocarditis (IE) [1] Furthermore, an association of *S. gallolyticus* subsp. *gallolyticus*-induced IE with colorectal malignancies has been observed in many cases [2–4]. Boleij et al. suggested a novel pathophysiological link between IE caused by *S. gallolyticus* subsp. *gallolyticus* and colorectal cancer, in which *S. gallolyticus* subsp. *gallolyticus* translocates through the adenomatous colon epithelium paracellular, followed by transport within the bloodstream towards the heart [5]. For IE pathogens, the adhesion to and/or invasion of endothelial cells of the endocardium is the first step in the establishment of vegetation. *S. gallolyticus* subsp. *gallolyticus* isolates have already been shown to have strain-dependent properties to adhere to or invade endothelial cells [6, 7].

Additionally, the interaction of *S. gallolyticus* subsp. *gallolyticus* with cells of the innate immune system plays an important role for survival in the host. It is initiated by bacterial pathogen-associated molecular patterns, which are detected by pathogen-recognition receptors to activate the innate immunity and counteract the infection [8]. Differences in virulence genes could provoke divergent strain-specific interactions with inflammatory cells and the induction of an inflammatory response during the transport of these bacteria within the bloodstream, potentially resulting in a significant difference in their ability to cause infection.

The pro-inflammatory cytokines, tumor necrosis factor-α (TNF-α), interleukin-1β (IL-1β) and IL-6, are the first secreted cytokines in a local infection, such as IE [9, 10]. Animal studies have suggested an intriguing role of IL-6 in the establishment of various infections [11–13]. Bustamante et al. showed an association between the inflammatory response, related to IL-6 and IL-8 cytokine profiles, and the outcome of an IE prosthetic valve endocarditis [14]. We, therefore, aimed to elucidate whether *S. gallolyticus* subsp. *gallolyticus* could provoke cytokine response in a strain-dependent manner from THP-1 monocytes and in a whole blood model. We reasoned that the potential of cytokine induction by different *S. gallolyticus* subsp. *gallolyticus* isolates could offer new insights into host-pathogen interaction. Because of the critical role of IL-6 in IE, the induction of IL-6 secretion was analyzed in whole blood. Monocytes are one of the first cells at an infection site and play a pivotal role in inflammatory response and recruitment of further immune cells [15–17]. Whereas IL-1β and TNF-α are the first cytokines in an infection and are generally pro-inflammatory [10], IL-6 is secreted thereafter and acts in both pro- and anti-inflammatory roles [18]. IL-8 and monocyte chemoattractant protein-1 (MCP-1) belong to the chemokine family and recruit more monocytes and other immune cells to the site of infection [19]. Thus, we analyzed the stimulation of expression of the IL-1β and IL-6 genes, and subsequently, that of IL-8, by *S. gallolyticus* subsp. *gallolyticus,* in THP-1 monocytes. Additionally, the survival of *S. gallolyticus* subsp. *gallolyticus* was also analyzed in this model.

Platelet aggregation in an IE acts like a double-edged sword; on one hand, platelets are anti-infective, releasing antimicrobial peptides and inflammatory mediators [20], and on the other hand, aggregated platelets and fibrin mask bacteria at the site of infection, which protects them from other immune cells in the tissue and blood [21]. The interaction of pathogens with platelets has been described as a virulence factor, and several pathogens are well-characterized for their relative potential to induce the aggregation of platelets [21–23]. Veloso et al. revealed that *S. gallolyticus* subsp. *gallolyticus* induces platelet aggregation rapidly, compared to *Enterococcus feacalis* [24]. Regarding the potential of bacteria to induce platelet aggregation as a virulence factor, our study has focused on determining whether strain-dependent differences in the induction potential can be revealed.

Finally, macrophages play a key role in innate immunity during an IE. Five percent of the surface of heart valve tissues infected by *Streptococcus* species is covered with macrophages [17]. It has already been shown that the *S. gallolyticus* subsp. *gallolyticus* isolate (UCN34) could survive much longer in macrophages compared to other bacteria (e.g. *Bacillus subtilis*) [5]. In the present study, we analyzed and compared the phagocytosis by macrophages and the survival of different *S. gallolyticus* subsp. *gallolyticus* strains in a cell culture model.

## Methods

### Cell culture and bacterial strains

THP-1 cells (ATCC, Wesel, Germany) were cultivated in Dulbecco’s modified Eagle’s medium (DMEM, Thermo Scientific, Waltham, USA) supplemented with 10% fetal calf serum (FCS, Pan Biotech, Aidenbach, Germany) and antibiotic/antimycotic solution (AB/AM, PAA Laboratories, Cölbe, Germany) at 37 °C and 5% CO_2_. *S. gallolyticus* subsp. *gallolyticus* strains, and *Staphylococcus aureus* for comparison (Table 1), were grown overnight in brain-heart infusion broth (Oxoid, Wesel, Germany) at 37 °C in a rotating shaker at 220 rpm. The bacterial titer was determined by serial dilutions in Dulbecco’s phosphate-buffered saline (DPBS) and plating 100 μl of an appropriate dilution on tryptone soya agar (Oxoid, Wesel, Germany) in triplicates for colony count.

**Table 1:** Bacterial strains: *S. gallolyticus* subsp. *gallolyticus* strains and *Staphylococcus aureus*

Species	Strain	Source	Origin
*S. gallolyticus* subsp. *gallolyticus*	DSM16831	animal	DSMZ
*S. gallolyticus* subsp. *gallolyticus*	DSM13808	sapropel	DSMZ
*S. gallolyticus* subsp. *gallolyticus*	isolate 010672/01	human, IE patient	HDZ, Germany
*S. gallolyticus* subsp. *gallolyticus*	Isolate 021702/06	human, IE patient	HDZ, Germany
*S. gallolyticus* subsp. *gallolyticus*	BAA-2069	human, IE patient	HDZ, Germany
*S. gallolyticus* subsp. *gallolyticus*	AC1181	human	RWTH Aachen
*S. gallolyticus* subsp. *gallolyticus*	AC6827	human	RWTH Aachen
*S. gallolyticus* subsp. *gallolyticus*	LMG17956	animal	Netherlands
*S. gallolyticus* subsp. *gallolyticus*	UCN34	human, IE patient	Calvados, France [50]
*S. aureus*	ATCC25923	Human	ATCC [51]

*DSMZ* German Collection of Microorganisms and Cell cultures, *RWTH Aachen* RWTH Aachen University, *LMG* Laboratory of Microbiology, Ghent University, *HDZ* Heart and Diabetes Center NRW, *ATCC* American Type Culture Collection

### Whole blood assay and measurement of IL-6

Inoculation of whole blood with *S. gallolyticus* subsp. *gallolyticus* strains was performed with human blood from three healthy volunteers. Blood was collected in heparin-gel monovettes (Kabe, Nümbrecht-Elsenroth, Germany) and mixed with RPMI1640 with or without *S. gallolyticus* subsp. *gallolyticus* from overnight cultures (initial bacterial titer: 1.6–4.8 × 10^5^ colony-forming units (CFU)/ml; volume ratio 1:10), as described previously [25]. Suspensions were incubated in 6-well plates at 37 °C and 5% CO_2_. Aliquots of 500 μl samples from either bacterial, blood or medium suspensions were used for the plating assay after 0, 6, 24 and 48 h. The experiment was performed on two different days with three technical replicates/day/volunteer. The residual probes were centrifuged at 1000 × *g* for 5 min and the supernatant was used for the measurement of IL-6 with a human IL-6 enzyme-linked immunosorbent assay (ELISA; Thermo Scientific, Waltham, USA), following the manufacturer’s instructions. Cell viability of leukocytes in whole blood was determined by flow cytometry (FACSCanto II; BD Biosciences, San José, USA) after lysis of the erythrocytes with BD Viaprobe (1:200).

### Quantification of inflammatory response of THP-1 cells upon infection by *S. gallolyticus* subsp. *gallolyticus*

THP-1 cells were counted with a Neubauer chamber and adjusted to 2 × 10^6^ cells/ml in DMEM with 10% FCS. Overnight cultures of bacteria were washed and diluted to approximately 10^5^ CFU/ml in DMEM and an aliquot was used for counting bacteria by plating and CFU assay. The cells were either inoculated with *S. gallolyticus* subsp. *gallolyticus* strains, with lipoteichoic acid (LTA, Sigma Aldrich, Darmstadt, Germany) as a positive control, or with DMEM medium as a negative control for 6 h. Afterwards, an aliquot of 100 μl was used for counting bacteria by plating assay. As before, the experiment was performed at 3 different days, with three technical replicates per day. For gene expression analysis, 2 ml sample was centrifuged at 10,000 rpm for 5 min and the THP-1 cell pellet was lysed with 600 μl RA1 buffer of the NucleoSpin RNAII kit (Marchery-Nagel, Düren, Germany).

### Gene expression analysis of THP-1 cells

Isolation of RNA was performed with the NucleoSpin RNA II kit, following the manufacturer’s instructions. The RNA was eluted with 25 μl RNase-free water and quantified using the NanoDrop 2000 (PeqLab, Erlangen, Germany). For the synthesis of cDNA, 2 μg RNA were diluted in 20 μl RNase-free water and cDNA synthesis was performed using the Superscript II Reverse Transcriptase Kit (Invitrogen, Carlsbad, USA), following the manufacturer’s instructions. The cDNA was used for relative quantitative real-time PCR. Real-time DNA amplification was measured on the Mastercycler ep Realplex platform (Eppendorf, Hamburg, Germany) using the Platinum SYBR Green qPCR SuperMix-UDG (Invitrogen, Carlsbad, USA). A volume of 10 μl was used for each reaction that contained 2.5 μl cDNA (dilution 1:5), 0.25 μl each Primer (20 μM), 5.0 μl Platinum SYBR Green reaction mix and 2.0 μl water, and three replicates were run per sample. Cycling conditions were as follows: degradation of uracil containing DNA at 50 °C for 120 s, initial denaturation at 95 °C (120 s), 45 cycles consisting of denaturation for 10 s at 95 °C, annealing at 58 °C for 15 s, and elongation at 72 °C for 20 s. Additionally, a melting curve served as a control for PCR amplification. Relative gene expression was calculated by normalization to hypoxanthine phosphoribosyltransferase 1, and glyceraldehyde-3-phosphate dehydrogenase by the efficiency-corrected ΔΔct method [26]. Sequences of the intron-spanning oligonucleotides for the reference genes hypoxanthine phosphoribosyltransferase 1 and glyceraldehyde-3-phosphate dehydrogenase and the genes of interest (*IL1B*, *IL6* and *IL8*) are listed in Table 2.

**Table 2:** Sequences of oligonucleotides used for relative quantification in real-time PCR

Gene name	*Forward* primer (5′ to 3′) *Reverse* primer (5′ to 3′)
Interleukin-1β (*IL1B*),	ACAGATGAAGTGCTCCTTCCA
	GTCGGAGATTCGTAGCTGGAT
Interleukin-6 (*IL6*)	ACAGCCACTCACCTCTTCAG
	GTGCCTCTTTGCTGCTTTCAC
Interleukin-8 (*IL8*)	GAACTGAGAGTGATTGAGAGTGGA
	CTCTTCAAAAACTTCTCCACAACC
Hypoxanthine phosphoribosyltransferase 1 (*HPRT*)	GCTGACCTGCTGGATTAC
	TGCGACCTTGACCATCTT
Glyceraldehyde-3-phosphate dehydrogenase (*GAPDH*)	AGGTCGGAGTCAACGGAT
	TCCTGGAAGATGGTGATG

### Platelet aggregation assay

Platelet aggregation was performed with platelet-rich plasma (PRP) of three healthy volunteers by light transmission aggregometry using the BORN method [27]. Blood was collected in PFA monovettes. The PRP was achieved by centrifugation (250 × *g*, 15 min without a break). Platelet-poor plasma was achieved by centrifugation of the monovettes at 2770 × *g* for 5 min. The PRP was diluted up to 200,000 platelets/μl within DPBS. Adjustment of the bacterial concentration was performed using the BactiFlow (bioMérieux, Marcy-l’Étoile, France), as described previously [28]. Suspensions (bacteria:platelet = 2:1) were used for aggregation in the Apact 4 f aggregometer (DiaSys, Holzheim, Germany). Acquisition was performed over 1800 s and changes of light transmission were acquired with the Apact Software. The experiment was performed on three different days with three technical replicates/day/volunteer.

### Phagocytosis assay

The macrophage assay was based on the phagocytosis assay of Boleij et al. [5] and Kaneko et al. [29]. In brief, THP-1 cells (5 × 10^5^ cell/well) were cultivated in 24-well plates with 50 ng/ml PMA in cell culture medium, which promoted macrophage differentiation in 3 days. On the third day, the medium was replaced with PMA-free medium after washing the cells twice with DPBS (Thermo Scientific, Waltham, USA). An overnight culture of *S. gallolyticus* subsp. *gallolyticus* was serially diluted (10^3^ dilution; 10^5^ CFU/ml) in DMEM supplemented with 10% FCS without AB/AM and the final bacterial titer of the inoculum was determined by plating assay. After washing the macrophages with DPBS three times, the *S. gallolyticus* subsp. *gallolyticus* dilution was added and plates were centrifuged at 400×*g* for 5 min to assure attachment of the bacteria to the macrophages. Phagocytic uptake of bacteria was ensured for 30 min at 37 °C and 5% CO_2_, and the macrophages were washed three times with DPBS. DMEM, including 10% FCS, 1 × AB/AM and 200 μg/ml gentamicin, was added for at least 20 min to kill the residual extracellular bacteria, and this was considered the 0 h time point [13]. At this time point (*t* = 0 h), all vital intracellular bacteria are captured. Additionally, the bacterial survival was determined at eight different sampling points (5, 8, 12, 16, 24, 30, 36 and 48 h). For each time point, the antibiotic-supplemented DMEM-medium was washed away, macrophages were lysed with 1% saponin and the bacterial titer determined by plating assay. The percentage of bacteria surviving after phagocytosis, was calculated relative to the initial phagocytized bacterial titer (at t = 0 h). The experiment was performed at two different days with four technical replicates per day.

### Measurement of cytotoxicity by lactate dehydrogenase release assay

*S. gallolyticus* subsp. *gallolyticus*-mediated cytotoxicity to THP-1 macrophages was detected 5 h after phagocytosis by measuring the lactate dehydrogenase (LDH) activity in the supernatant. A quantity of 1.3 × 10^5^ THP-1 cells were seeded in each well of a 96-well plate in 100 μl DMEM supplemented with 10% FCS, and AB/AM and differentiation was carried out as described above. The Pierce LDH Assay Kit (Thermo Scientific, Waltham, USA) was used for this analysis. The analysis was carried out following the manufacturer’s instructions. The amount of LDH was determined in a Reader Infinite® m200 PRO microplate reader (Tecan, Männedorf, Switzerland).

### Statistics

Experimental data were analyzed by Mann-Whitney U test using GraphPad Prism 6.0 (GraphPad Software). *P* < 0.05 was considered statistically significant. Mean with standard error is displayed in figures.

## Results

### Bacterial growth of *S. gallolyticus* subsp. *gallolyticus* isolates in a whole blood model

As indicated earlier, *S. gallolyticus* subsp. *gallolyticus* must survive in the bloodstream in order to reach the endocardium. To elucidate the survival of different *S. gallolyticus* subsp. *gallolyticus* strains in whole blood, the bacterial titers were determined after 6, 24 and 48 h of inoculation.

**Fig. 1:**
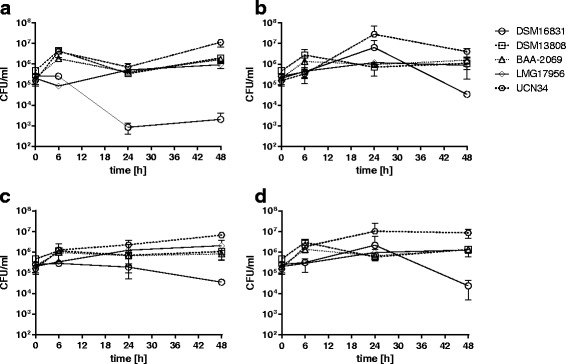
Growth of *S. gallolyticus* subsp. *gallolyticus* strains in a whole blood model. Bacterial growth (with mean and standard deviation) of the *S. gallolyticus* subsp. *gallolyticus* strains DSM16831, DSM13808, BAA-2069, LMG17956 and UCN34 was determined in a whole blood model with blood from three different volunteers (n = 2 per volunteer), as described in Materials and Methods. The bacterial titers (CFU/ml) in each volunteer blood (**a-c**) and the merged (averaged) values of all (**d**) are displayed as indicated. Bacterial titer was determined by plating assay

We did not include intermediate time points (8 h and 30 h) in our routine assays, since they added no additional information regarding the bacterial survival kinetics (data not shown). The cell viability was determined by flow cytometry. About 70% of the leukocytes were still viable regardless of which sample was observed. The bacterial growth was found to be influenced by the blood of the different volunteers and differed between the isolates analyzed (Fig. 1). Specifically, the bacterial titer of strain DSM16831 decreased continuously in the whole blood of volunteer 1 (Fig. 1a). After 48 h of incubation (2 × 10^3^ CFU/ml), the titer of volunteer 1 was still low, while the titer of this same isolate in the whole blood of the other volunteers was about ten times higher (3.5 × 10^4^ CFU/ml; Fig. 1b and c). For example, proliferation of DSM16831 was observed in the whole blood of volunteer 2 up to 24 h, but a persistent bacterial titer was noticed in the whole blood of volunteer 3.

The comparison of strains revealed that strain UCN34 could grow in whole blood during 48 h of incubation (Fig. 1d). In the volunteer 1 blood, UCN34 grew in the first 6 h of incubation, the titer then decreased for the next 18 h, and increased again up to 4.1 × 10^6^ CFU/ml at 48 h (Fig. 1a). In the blood of volunteer 2, UCN34 grew in the first 24 h (titer increased by a factor of 100), but the titer decreased after 48 h (Fig. 1b). In contrast, a continuous growth of this strain (0 h: 1.6 × 10^5^ CFU/ml; 48 h: 6.7 × 10^6^ CFU/ml) was observed in the whole blood of volunteer 3 (Fig. 1c). In Fig. 1d, we summarize the survival or growth of all strains in whole blood of all three volunteers. As seen, the titer of the strains DSM13808, BAA-2069 and LMG17956 in all three volunteers blood lied between the titer of the strain UCN34 and the titer of DSM16831 after 48 h of incubation (Fig. 1d). The initial titer of these isolates was 2–4.8 × 10^5^ CFU/ml, which increased only slightly by a factor of about 10 within 48 h.

### Induction of IL-6 secretion by immune cells in whole blood after *S. gallolyticus* subsp. *gallolyticus* inoculation

**Fig. 2:**
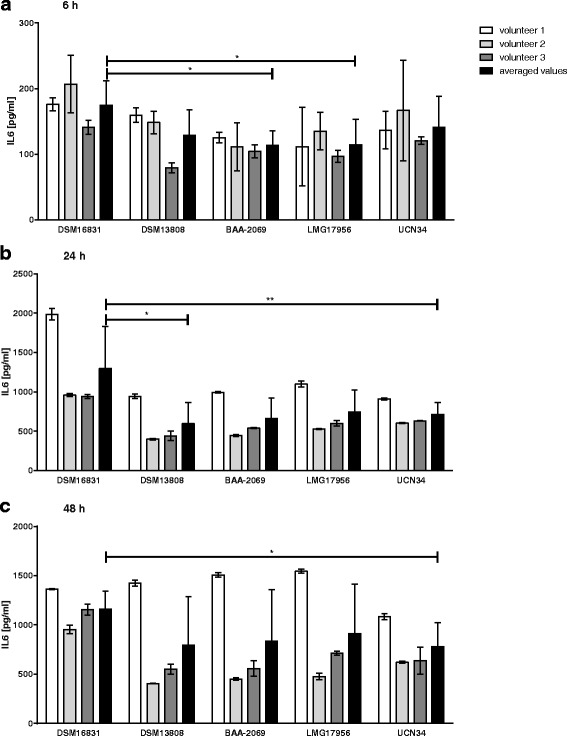
Induction of IL6 in a whole blood model of *S. gallolyticus* subsp. *gallolyticus* strains. IL6 concentration (mean and standard deviation) after inoculation for 6 (**a**), 24 (**b**) and 48 h (**c**) with the *S. gallolyticus* subsp. *gallolyticus* strains DSM16831, DSM13808, BAA-2069, LMG17956 and UCN34 in a whole blood model of three different volunteers (*n* = 2 per volunteer), determined as described in Materials and Methods. The averaged IL6 concentration of all three volunteers (black bars) was used to demonstrate significant differences between the isolates (Mann-Whitney test, **: p ≤ 0.005; *: p ≤ 0.05)

The survival of bacteria in whole blood is influenced by the inflammatory response of immune cells. Upon measuring the bacterial survival, we quantified the IL-6 concentration in whole blood supernatants, based on the premise that this will reflect the ability of different *S. gallolyticus* subsp. *gallolyticus* isolates to regulate the inflammatory response of the host, which may be relevant to bacterial escape of the innate immune system. Results (Fig. 2) show that after 6 h of incubation with the bacteria, only marginal divergences could be observed among the different study volunteers. Stronger individual variations were seen after 24 and 48 h incubations. Specifically, IL-6 induction in volunteer 1 was higher overall compared to volunteers 2 and 3, independent of the *S. gallolyticus* subsp. *gallolyticus* isolate used for the inoculation. Volunteer 2 elicited the lowest IL-6 expression after 24 and 48 h. The averaged IL-6 values of all three individuals were then used to compare the strain-dependent induction of IL-6 synthesis (Fig. 1b). At 6 h post-incubation, the *S. gallolyticus* subsp. *gallolyticus* strain DSM16831 induced slightly higher amounts of IL-6 than the other strains. The capability of DSM16831 to induce IL-6 in whole blood was distinctly higher than that of BAA-2069 and LMG17956 (Fig. 1a; 175 pg/ml vs. 114 pg/ml). At 24 h, IL-6 secretion elicited by DSM16831 was also the highest (Fig. 2b), being about twice as high as those observed for the four other strains (e.g. volunteer 2: DSM16831: 959 pg/ml, DSM13808: 400 pg/ml, BAA-2069: 445 pg/ml and UCN34: 602 pg/ml). A decrease in the IL-6 concentration from 24 to 48 h of incubation of DSM16831 in the whole blood of volunteer 1 was also noted (Fig. 2c). By contrast, incubation with the other isolates and in the other volunteers showed an increase of the IL-6 concentration after 48 h compared to 24 h. Taken together, these results revealed inter-individual differences as well as strain-dependency in IL-6 stimulation.

### Induction of *IL1B*, *IL6* and *IL8* gene expressions in THP-1 monocytes by *S. gallolyticus* subsp. *gallolyticus* strains

**Fig. 3:**
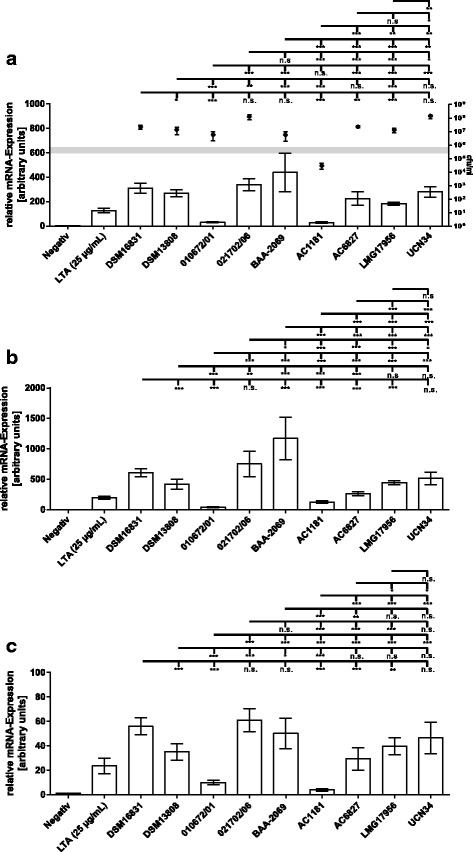
Bacterial titer and induction of gene expression of *IL1B* (**a**), *IL6* (**b**) and *IL8* (**c**). THP-1 cells were inoculated with 2.4–7.1 × 10^5^ CFU/ml of *S. gallolyticus* subsp. *gallolyticus* strains. After 6 h of inoculation, the bacterial titer was determined by plating assay in triplicate (*n* = 3) and displayed as black dots in panel A. The bacterial titers are shown on the right-hand y-axis, and the horizontal grey line represents the input titer. LTA (25 μg/ml) and media were used as positive and negative controls, respectively (bars; n = 3). After 6 h of inoculation, the relative gene expression of *IL1B*, *IL6* and *IL8* was determined using real-time PCR. Comparison of induction between the isolates (lines above the bars) was performed using the Mann-Whitney test (n.s: not significant, *: *p* ≤ 0.05, **: *p* ≤ 0.005, ***: *p* ≤ 0.0005)

Since monocytes are early-response immune cells that are recruited to the site of infection in IE and play an important role in inflammation, one can argue that *S. gallolyticus* subsp. *gallolyticus* would benefit from a reduced recruitment of monocytes and lower induction of inflammatory cytokines and chemokines. To test this in the absence of any influence of individual factors in blood, we used a cell culture model (THP-1 monocytes) and analyzed the stimulation potential of different *S. gallolyticus* subsp. *gallolyticus* strains. THP-1 cells were inoculated with 2.4–7.1 × 10^5^ CFU/ml *S. gallolyticus* subsp. *gallolyticus* strains for 6 h. We first determined the bacterial titer to exclude a potential difference in the stimulation of cytokine gene expression due to divergence in bacterial growth. As shown (Fig. 3a; right y-axes), 8 *S. gallolyticus* subsp. *gallolyticus* isolates grew to 5.7 × 10^6^–1.5 × 10^8^ CFU/ml after 6 h of incubation. Only the bacterial titer of strain AC1181 decreased during the 6 h of incubation to 3 × 10^4^ CFU/ml.

We then quantified the expression of *IL1B*, *IL6* and *IL8* genes after induction with different *S. gallolyticus* subsp. *gallolyticus* isolates, using LTA (25 μg/ml) as positive control. Results (Fig. 3a-c) show that the induction of all three genes after inoculation with the *S. gallolyticus* subsp. *gallolyticus* strains AC1181 and isolate 010672/01 was lower compared to the induction by other strains. By contrast, the isolates DSM16831, 021702/06 and BAA-2069 induced 10-fold higher gene expression of *IL1B, IL6* and *IL8*. The relative gene expression levels (arbitrary units: au) were: *IL1B*: DSM16831: 311, 021702/01: 340, BAA-2069: 440, 010672/01: 31, AC1181: 28; *IL6*: DSM16831: 608, 021702/01: 753, BAA-2069: 1172, 010672/01: 42, AC1181: 128; *IL8*: DSM16831: 56, 021702/01: 61, BAA-2069: 50, 010672/01: 10, AC1181: 4.

### Induction of platelet aggregation by *S. gallolyticus* subsp. *gallolyticus*

**Fig. 4:**
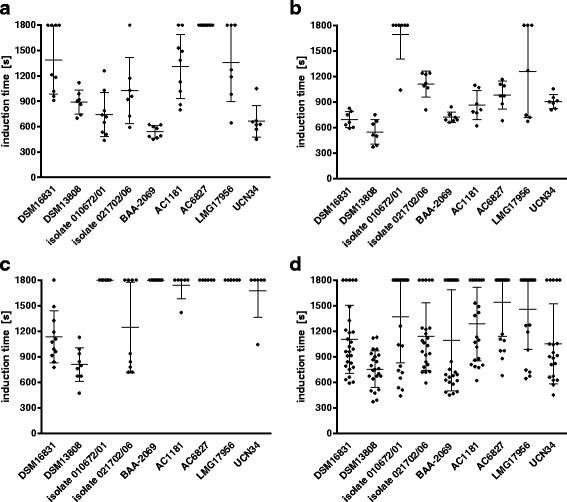
Induction time of platelet aggregation with *S. gallolyticus* subsp. *gallolyticus* strains. The PRPs of three volunteers (**a**-**c**) were inoculated with the indicated *S. gallolyticus* subsp. *gallolyticus* strains. The induction times of the strains to promote platelet aggregation in all three volunteers are summed up in panel **d**. The time to induce a detectable platelet aggregation was determined using light transmission aggregation (BORN method; n = 3), as described in Materials and Methods. Value obtained with a sample in which no platelet aggregation was induced is listed and set at 1800 s

As mentioned earlier, platelet aggregation is a virulence factor of different *Streptococcus* species, and it was shown that *S. gallolyticus* subsp. *gallolyticus* promotes platelet aggregation as well [24]. To determine the inter-individual variations of this property, we examined the induction of platelet aggregation by PRP of the same three volunteers that were recruited in the whole blood study, using light transmission aggregometry as described in Materials and Methods. The results (Fig. 4) show the comparison of strains for each volunteer (Fig. 4a-c) and averaged values of platelet aggregation of all three volunteers (Fig. 4d). It can be seen that the distribution of the summed values of all volunteers is very high in (Fig. 4d). Isolate 010672/01, for example, induced platelet aggregation during the inoculation of PRP of volunteer 1 after 741 s (Fig. 4a). By contrast, aggregation of platelets from volunteers 2 and 3 was not induced within 1800 s with this isolate (Fig. 4b and c). Similarly, isolate AC6827 induced aggregation of platelets of volunteer 2 only (Fig. 4b). It is also noteworthy that the platelets of volunteer 3 only aggregated after stimulation with the isolates DSM16831, DSM13808 and 021702/06 (Fig. 4c), while the other six isolates could not induce platelet aggregation. Clearly, the potential of *S. gallolyticus* subsp. *gallolyticus* to induce platelet aggregation depends highly on individual host factors.

Regardless of host specificity, the induction of platelet aggregation with the *S. gallolyticus* subsp. *gallolyticus* isolates AC6827 and LMG17956 was modest overall in all volunteers (Fig. 4d). By contrast, isolate DSM13808 induced platelet aggregation of the platelets of all three volunteers comparably rapidly after the start of inoculation (Fig. 4a-d; volunteer 1: 890 s, volunteer 2: 547 s, volunteer 3: 808 s). The isolate BAA-2069 showed comparably quick induction of platelet aggregation of volunteer 1 and 2, while no induction of platelet aggregation was determined in the PRP of volunteer 3 (Fig. 4a-c).

### Strain-dependent phagocytosis of *S. gallolyticus* subsp. *gallolyticus* by macrophages

**Fig. 5:**
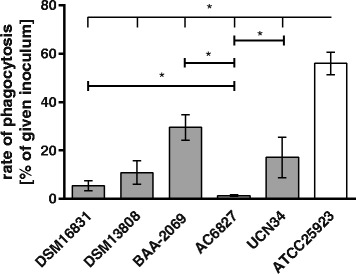
Rate of phagocytosis of *S. gallolyticus* subsp. *gallolyticus* strains by THP-1 macrophages. Phagocytosis rates (shown with mean and standard error) for the indicated strains are expressed as percentage of the inoculum, incubated for 30 min (n = 2). Significant differences between the isolates were calculated by the Mann-Whitney test (*: p ≤ 0.05; GraphPad Prism 6.0). The grey bars represent the survival of *S. gallolyticus* subsp. *gallolyticus* strains, and the white bar, that of *Staphylococcus aureus* (ATCC25923)

Following survival in the human blood stream, *S. gallolyticus* subsp. *gallolyticus* needs to survive in the tissue of the endocardium. In this context, by far the most important cells of the innate immune system are the macrophages [17]. To analyze the clearance of *S. gallolyticus* subsp. *gallolyticus* by macrophages, the cells of the THP-1 macrophage line were inoculated with five different *S. gallolyticus* subsp. *gallolyticus* isolates and the *Staphylococcus aureus* strain ATCC25923 (Fig. 5). This methicillin-sensitive *Staphylococcus aureus* isolate is used as a strain of comparison in different models of infectious diseases [30–32].

The number of phagocytized bacteria varied significantly between the *S. gallolyticus* subsp. *gallolyticus* isolates. Strain DSM16831 (5.41%), DSM13808 (10.9%) and UCN34 (17.2%) were phagocytized by almost the same amount. The strain BAA-2069 was phagocytized significantly higher (BAA-2069: 29.5%) by THP-1 macrophages, whereas only 1.4% of the inoculum of AC6827 given was found in the macrophages. All *S. gallolyticus* subsp. *gallolyticus* isolates were less phagocytized than *Staphylococcus aureus.*

**Fig. 6:**
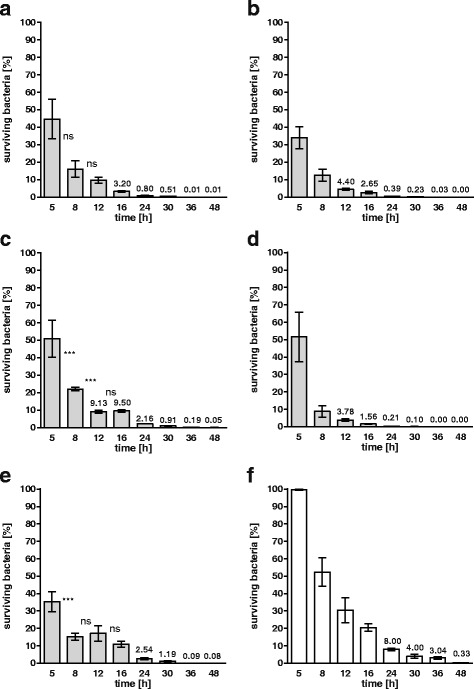
Survival of *S. gallolyticus* subsp. *gallolyticus* strains in macrophages. Percentage of surviving bacteria after 48 h of phagocytosis is shown for DSM16831 (**a**), DSM13808 (**b**), BAA-2069 (**c**), AC6827 (**d**) and UCN34 (**e**) and the *Staphylococcus aureus* strain ATCC25923 (**f**) (mean with standard error, relative to the bacterial titer at *t* = 0 h). The grey bars represent *S. gallolyticus* subsp. *gallolyticus* strains and the white bars represent *Staphylococcus aureus* (n = 2)

The bacterial titer was determined at different time points within 48 h to investigate whether bacteria were destroyed inside the macrophages. The survival of the *S. gallolyticus* subsp. *gallolyticus* isolates and of the *Staphylococcus aureus* isolate ATCC25923 is shown in Fig. 6. All *S. gallolyticus* subsp. *gallolyticus* isolates and the *Staphylococcus aureus* isolate were continuously eliminated, however, only the isolates BAA-2069 and UCN34 showed a constant titer between 12 and 16 h or 8 and 16 h.

## Discussion

This study elucidated strain-dependent divergences regarding several potential pathogenic pathways of *S. gallolyticus* subsp. *gallolyticus*-induced infections, the results of which can provide the basis for future studies of pathogenesis by this bacterium and its various strains. Through systematic review and meta-analysis, Boleij et al. (2011) proposed that *S. gallolyticus* subsp. *gallolyticus* translocates paracellularly through malignant colonic lesions into the bloodstream [5]. We reasoned that the pathomechanisms of the process must involve survival of the bacteria within the bloodstream as well as adherence, persistence and proliferation at the endocardium [33]. Thus, we initiated a comprehensive analysis relevant to various steps of the process. To summarize, we first analyzed bacterial growth and survival in a whole blood model. We then focused on the response of immune cells to different *S. gallolyticus* subsp. *gallolyticus* isolates, whereby we quantified the protein and mRNA of strategic cytokines and chemokines. We also studied platelet aggregation, since in addition to survival against innate immunity, induction of platelet aggregation plays a major role in bacterial colonization at the endocardium as it masks the pathogen against immune cells of the human blood [34].

We have shown that the growth characteristics in the whole blood model differed between the *S. gallolyticus* subsp. *gallolyticu*s isolates. Difference in cell viability in blood, stimulated by different *S. gallolyticus* subsp. *gallolyticus* strains, was not observed by flow cytometric analysis, but *S. gallolyticus* subsp. *gallolyticus-*stimulated blood showed a reduction of granulocytes compared to the control with no bacteria. This is due most probably to phagocytic uptake by and apoptosis of these cells [35, 36].

In our analysis of the inflammatory response of THP-1 monocytes to different *S. gallolyticus* subsp. *gallolyticus* strains, we analyzed the gene expression of the early cytokine TNF-α and the later chemokine MCP-1, whereby the same expression patterns were detected as shown for the genes *IL1β, IL6* and *IL8* (data not shown). Stimulation of THP-1 monocytes with the isolates DSM16831 and BAA-2069 led to the highest increase in expression of the *IL1B*, *IL6* and *IL8* genes, whereas the isolates 010672/01 and AC1181 led only to a marginal increase in gene expression of these cytokines. Although we do not know the exact reason for the difference, at least in the case of AC1181, the lower response can be due to a dose-effect relationship. Specifically, in studies of the bacterial titer in the presence of THP-1 monocytes, AC1181 showed a decrease in the multiplicity of infection after 6 h of incubation, which could be potentially due to phagocytosis and killing through monocytes [37]. In case of the isolate 010672/01, however, the induction of the cytokine gene expression was low even though the titer was comparable to those of the other strains.

There is evidence that the absence of IL-6 in the early stage of an IE leads to a higher mortality in mouse [13]. Moreover, it was also shown that a decrease in IL-6 secretion leads to a higher survival of mice with sepsis [12]. Our data confirmed that IL-6 secretion in *S. gallolyticus* subsp. *gallolyticus* infection depends on both the strain and the individual blood. As summarized in Table 3, DSM16831 caused an early immune response followed by an early elimination in whole blood, whereas UCN34 could grow without a strong activation of the immune system. It is likely that the difference is due to different surface proteins and teichoic acids that may be involved in bacteria-host recognition and resultant signaling, such that DSM16831 is more efficiently recognized by the host immune cells, leading to a higher immune response and more rapid bacterial killing. Evidently, an analysis of the bacterial surface in these strains may lead to a better understanding of the recognition process and the resultant phenotypes.

**Table 3:** Summarized results of the blood assay and platelet aggregation. The outcome is scaled from killed bacterial cells, low IL-6 secretion by blood cells and no stimulation of platelet aggregation to growth in blood, high IL-6 secretion and fast platelet aggregation by these symbols: -−−/−−/−/0/+/++/+++

		Volunteer 1	Volunteer 2	Volunteer 3
DSM16831	Blood survival	–-	0	0
	IL6 conc. (48 h)	+++	++	++
	Aggregation	–	+	–
DSM13808	Blood survival	+	+	+
	IL6 conc. (48 h)	+	0	0
	Aggregation	0	+	0
BAA-2069	Blood survival	+	+	+
	IL6 conc. (48 h)	+	0	0
	Aggregation	+	+	–--
LMG17956	Blood survival	+	+	+
	IL6 conc. (48 h)	+	0	0
	Aggregation	–-	–-	–--
UCN34	Blood survival	++	++	++
	IL6 conc. (48 h)	+	0	0
	Aggregation	+	0	–--

In addition to the survival in blood, persistence and colonization at the endocardium are required for successful infection [33]. Survival in phagocytic cells (such as macrophages) and the platelet aggregation can, on one hand, act bactericidally, but on the other hand, may also mask the bacteria by forming vegetation. For this reason, we have studied both processes in multiple bacterial isolates. As we have shown, the rate of phagocytosis of DSM16831 was low; additionally, this strain was degraded continuously in macrophages, which is in contrast to the strains BAA-2069 and UCN34. The phenotype of DSM16831 implies overall low virulence, since DSM16831 was also not able to invade endothelial cells in vitro in contrast to many other *S. gallolyticus* subsp. *gallolyticus* strains tested [7]. The ability of UCN34 and BAA-2069 to survive in macrophages for a certain time clearly presents a potentially important advantage. In fact, this phenotype has already been determined as a high virulence characteristic for *Staphylococcus aureus* and *S. mutans* [38, 39].

De Herdt et al. (1995) observed intracellular replication of distinct *S. bovis* strains after phagocytosis by macrophages from pigeons, which also induced lysis of all macrophages after 7 h of incubation [40]. We did not observe this phenotype of *S. bovis* in the primary macrophages for *S. gallolyticus* subsp. *gallolyticus* in the macrophage-like THP-1 cells, as only 20% of the macrophages were lysed after 24 h, regardless of whether the strain was highly or modestly phagocytized. While persisting in macrophages and endothelial cells [7], the *S. gallolyticus* subsp. *gallolyticus* strains UCN34 and BAA-2069 could have an advantage over other strains in, for example, antibiotic treatment, resulting in better persistence in tissue and blood. Based on these results, we performed a transcriptome analysis of *S. gallolyticus* subsp. *gallolyticus* following phagocytosis by THP-1 macrophages. This revealed that *S. gallolyticus* subsp. *gallolyticus* reacts probably to oxidative burst with a higher gene expression of NADH oxidase directly after phagocytosis. Five hours after phagocytosis, the gene expression of Dlt-proteins, which are required for D-alanylation of teichoic acids, and proteins involved in carbohydrate metabolism and transport systems were upregulated [41]. Danne et al. (2014) presented a linkage between pili expression of the strain UCN 34 and phagocytosis, thereby showing a higher phagocytosis of highly piliated bacterial cells. In addition, genotypic differences regarding the *pilB* gene between strains could also influence IL-6 induction and survival in whole blood [42]. Interestingly, DSM16831 and AC6827 lack the gene *pilB* and also show a weak intracellular uptake through phagocytosis [7]. This correlation should be analyzed with other strains with and without the *pilB* gene to understand the mechanism of differences in bacterial uptake by phagocytosis.

In this study, we have clearly documented high individual variances in different *S. gallolyticus* subsp. *gallolyticus* isolates to induce platelet aggregation. While DSM13808 led to considerably higher aggregation, the isolates LMG17956 and AC6827 were weaker agents (Table 3). Conceivably, such induction potential can be highly relevant to the pathogenicity of strains for causing IE in vivo. In corollary, mediators of platelet aggregation by *S. gallolyticus* subsp. *gallolyticus* can be considered virulence factors in IE. Future studies may reveal the identity of the factors, but it is tempting to speculate that the interactions between platelets and *S. gallolyticus* subsp. *gallolyticus* is mediated by platelet receptors, such as GPIb or GPIIb/IIIa, as was shown for other bacteria, such as *Staphylococcus aureus* or *Streptococcus sanguinis* [23, 43].

*S. gallolyticus* subsp. *gallolyticus* strains show very different characteristics in survival and stimulation potential with regard to human blood cells. Unfortunately, a direct comparison between human pathogenic isolates and those from non-human sources is not possible. Nevertheless, it is worth noting that the human IE isolate UCN34 showed a high virulent phenotype whereas the strain DSM16831 from koala feces showed a low virulent phenotype [7]. On the other hand, DSM13808, collected from sapropel (not associated with infection) led to fast platelet aggregation (high virulence factor) but had a moderate phenotype in other analyses, compared to other strains. Additionally, our results show comparable phenotypes between the strains BAA-2069, isolated from an IE-patient, and LMG17956, isolated from calf. However, BAA-2069 induced the aggregation of platelets earlier than LMG17956. Thus, genetic information, such as distinct sequence types revealed by multilocus sequence typing could not be correlated with observed phenotypes [44]. Because of these different characteristics in phenotypic experiments versus genotype of each strain, it is unfeasible to assign the origin to the strains methodically [7, 44]. This has also led to the hypothesis that *S. gallolyticus* subsp. *gallolyticus* has the potential for zoonosis and may transmit from animal to human and vice versa [45].

In addition to the bacterial strain-dependent influence, interindividual variations in responses to the bacteria also influence an IE. Genetic predispositions to IE and platelet aggregation have already been found [46, 47]. Individual host responses to the *S. gallolyticus* subsp. *gallolyticus* isolates were observed in this study in whole blood and platelet aggregation, whereby blood from the same volunteers was used for both assays for unambiguous comparison. The high inter-individual divergences of platelet aggregation observed was also described for stimulation by *Streptococcus sanguinis* [48]. Finally, platelet aggregation by pathogens is likely an important virulence factor that promotes IE establishment, and hence, it will be quite interesting to identify the host factors that underlie the relevant interactions [49].

## Conclusions

Our results presented here have contributed to uncover the nature of the phenotypes of *S. gallolyticus* subsp. *gallolyticus* strains in vitro*,* which may influence the infection process in vivo. The DSM16831 isolate remains particularly interesting, because of its very low virulent phenotype in vitro and likely poor survival in tissue and blood compared to the other isolates tested (e.g. UCN34 and BAA-2069). These findings will form the foundation for further research that should provide a more detailed knowledge of the pathogenicity factors of *S. gallolyticus* subsp. *gallolyticus* isolates that contribute to divergent phenotypes. The results should enhance our understanding of pathophysiological interrelationship between these factors and the establishment of IE that leads to congestive heart failure.

## Abbreviations

AB/AM: Antibiotic/antimycotic solution
Au: Arbitrary units
CFU: Colony-forming units
DPBS: Dulbecco’s phosphate-buffered saline
FCS: Fetal calf serum
IE: Infective endocarditis
IL: Interleukin
LDH: Lactate dehydrogenase
LTA: Lipoteichoic acid; PRP: platelet-rich plasma
